# Short, Synthetic Cationic Peptides Have Antibacterial Activity against *Mycobacterium smegmatis* by Forming Pores in Membrane and Synergizing with Antibiotics

**DOI:** 10.3390/antibiotics4030358

**Published:** 2015-08-24

**Authors:** Kajal Gupta, Sameer Singh, Monique L. van Hoek

**Affiliations:** 1 College of Sciences, George Mason University, Fairfax, VA 22030, USA; E-Mail: kgupta8@gmu.edu; 2 Thomas Jefferson High School for Science and Technology, Fairfax, VA 22312, USA; E-Mail: samster1243@gmail.com; 3 School of Systems Biology, George Mason University, Manassas, VA 20110, USA; 4 National Center for Biodefense and Infectious Diseases, George Mason University, Manassas, VA 20110, USA

**Keywords:** mycobacterium, cationic AMPs, antibiotics, synergy

## Abstract

Multicellular organisms are constantly exposed to a multitude of pathogenic microbes. Infection is inhibited *in vivo* by the innate and adaptive immune system. *Mycobacterium* species have emerged that are resistant to most antibiotics. We identified several naturally occurring cationic antimicrobial peptides that were active at low micromolar concentrations against *Mycobacterium smegmatis*. Human-derived cathelicidin LL-37 is well characterized and studied against *M. smegmatis*; we compared LL-37 with Chinese cobra-derived cathelicidin NA-CATH and mouse cathelicidin (mCRAMP). Two synthetic 11-residue peptides (ATRA-1A and ATRA-2) containing variations of a repeated motif within NA-CATH were tested for their activity against *M. smegmatis* along with a short synthetic peptide derivative from the human beta-defensin hBD3 (hBD3-Pep4). We hypothesized that these smaller synthetic peptides may demonstrate antimicrobial effectiveness with shorter length (and at less cost), making them strong potential candidates for development into broad-spectrum antimicrobial compounds or use in combination with antibiotics. These peptides have antimicrobial activity with EC_50_ ranging from 0.05 to 1.88 μg/mL against *Mycobacterium smegmati*s. The ATRA-1A short peptide was found to be the most effective antimicrobial peptide (AMP) (EC_50_ = 0.05 μg/mL). High bactericidal activity correlated with bacterial membrane depolarization and permeabilization activities. The efficacy of the peptides was further analyzed through Minimal Inhibitory Concentration (MIC) assays. The MICs were determined by the microdilution method. The peptide mCRAMP showed the best MIC activity at 15.6 μg/mL. Neither of the effective short synthetic peptides demonstrated synergy with the antibiotic rifampicin, although both demonstrated synergy with the cyclic peptide antibiotic polymyxin B. The peptides LL-37 and mCRAMP displayed synergism with rifampicin in MIC assays, whereas antibiotic polymyxin B displayed synergism with LL-37, ATRA-1A, and hBD3-Pep4. In further studies, polymyxin B synergized with LL-37, ATRA-1A, and hBD3-Pep4 while Rifampicin synergized with LL-37 and mCRAMP for intracellular killing of mycobacteria residing inside macrophages. These studies provide the foundation for the potential development of synthetic cationic antimicrobial peptides with activity against mycobacteria.

## 1. Introduction

Antimicrobial peptides (AMPs) are essential components of innate immunity in humans and other higher organisms, contributing to our first line of defense against infection [1], and are widespread and abundant in nature. The selectivity, potency, and effectiveness of AMPs depends on their physicochemical properties including their amphipathic nature, net charge, charge angle, overall hydrophobicity, and conformational flexibility [2,3]. AMPs can permeabilize membranes or form cytotoxic pores in bacterial membranes, but also inhibit cell wall, nucleic acid, and protein biosynthesis [4]. They have also been shown to be capable of binding and neutralizing lipopolysaccharides, promoting angiogenesis and wound healing, and exerting anti-tumor activity [5]. During mycobacterial infections, host defense peptides including cathelicidin, defensin, and hepcidin have antimicrobial activities against mycobacteria, making them promising candidates for future drug development [6].

The cathelicidin family is a large and diverse collection of cationic antimicrobial peptides (cAMPs) found in a variety of vertebrate hosts that range from 12 to 80 amino acid residues in length [7]. In humans, only one cathelicidin (LL-37) has been characterized [8]. This peptide is included as a control in the studies as it has previously been shown to be active against *M. smegmatis* [9].

Vertebrate defensins are cAMPs that contain three well-conserved intramolecular disulfide bonds and can be grouped into defensin sub-families based on functional and structural properties [10]. β-Defensins are predominantly expressed by epithelial cells; however, low-level expression of β-defensins has been observed in other organs. To date, the majority of research has focused on four human β-defensins, hBD1, -2, -3, and -4. These inducible host-defense peptides demonstrate broad spectrum antimicrobial effectiveness against Gram-positive and Gram-negative bacteria, fungi, and some enveloped viruses, with hBD3 demonstrating the broadest antimicrobial effectiveness, including activity against *Francisella (F.) tularensis* LVS and *F. novicida* [11].

Recently, short, synthetic peptides with sequences based on portions of the amino acid sequence of hBD3 were reported to exhibit varied antimicrobial potencies against *Escherichia coli* and *Staphylococcus aureus* [10]. We designed a synthetic, short decapeptide called Peptide 4 (hBD3-Pep4) that demonstrated significant antimicrobial potency based on the C-terminal portion of hBD3 [2]. In this peptide, two cysteine residues found in the full-length hBD3 peptide were substituted by two serine residues to eliminate the potential for disulfide bond formation, and the C-terminus was amidated to increase charge.

Mycobacteria are characterized by their unusual lipid-rich cell wall, composed of a variety of unique glycoconjugates and intercalating complex lipids, offering a highly impermeable barrier for common antibiotics. It is noteworthy that the mycolic acid outer layer provides a wax-like architecture to the cell wall that can hinder the uptake of many antimycobacterial drugs [12]. *M. tuberculosis* is often described as being CAMP-tolerant. Many natural CAMPs are active against *M. tuberculosis* only at rather high concentrations that are not practical for tuberculosis (TB) treatment [13]. Specific features of the antimicrobial peptides (AMPs), such as low molecular weight, high cationicity, amphipathic structure [9], immunomodulatory effects, and diverse modes of action [14], make them an interesting source of novel antimycobacterial agents [6]. Furthermore, CAMPs can be cytotoxic to mammalian cells, and long peptides are relatively expensive to produce. However, naturally occurring AMPs, including cathelicidin LL-37 and alpha-defensin human neutrophil peptides (HNPs), have been documented to kill *M. tuberculosis*, albeit at very high concentrations [15,16,17,18].

We have previously identified small synthetic peptides within the Chinese cobra (*Naja atra*) cathelicidin NA-CATH, based on an imperfect repeated 11-amino-acid motif (ATRA motif) [19]. We further modified the sequence to generate two small, synthetic antimicrobial peptides, ATRA-1A and ATRA-2 [19], and determined that ATRA-1A was generally highly effective against both Gram-negative [19,20,21] and Gram-positive [22] bacteria, while ATRA-2 was generally inactive.

We found that LL-37 and mCRAMP synergized with rifampicin in Minimal Inhibitory Concentration (MIC) assays. More interestingly, LL-37, ATRA-1A, mCRAMP, and hBD3-Pep4 showed synergy with the antibiotic polymyxin B. An infected macrophage model was used to test the intracellular activity of these peptides with and without synergy with antibiotics. The antimicrobial peptides tested in the study exert their activity by forming pores and thus permeabilizing the bacterial membrane.

In this work, novel small, synthetic antimicrobial peptides that we have previously developed were tested for activity against *M. smegmatis* as a first model of *Mycobacterium* spp. We assessed the efficiency of previously known antimicrobial peptides against *Mycobacterium* and studied the mode of action of each peptide. We further studied the synergic effect of these peptides with known antibiotics reported against *Mycobacterium*. Finally, we have shown the intracellular killing of *Mycobacterium* in J774A.1 macrophages by peptides and the synergy of peptides and antibiotics. Further studies will be done to test the most efficient peptides against *M. tuberculosis*.

## 2. Materials and Methods

### 2.1. Bacterial and Mammalian Cells

*M. smegmatis* mc^2^155 was grown in Middlebrook 7H9 (Difco) broth with 2% (*w*/*v*) glucose as the carbon source, and 0.05% (v/v) Tween 80, on shaker at 130 rpm. Cultures of *M. smegmatis* were stored at −80 °C. The CFU/mL was determined by growth on MB7H9 containing 1.5% (*w*/*v*) agar. For bactericidal assays, frozen enumerated aliquots were thawed immediately prior to use. Cell growth was monitored at O.D. 600 nm. The CFU/mL was determined with a standard curve of absorbance *vs.* CFU/mL. The mouse macrophage cell line J774A.1 (ATCC-TIB-67) was cultured in Dulbecco modified Eagle medium (DMEM; Life Technologies 11995073) supplemented with 10% fetal calf serum, 1% penicillin-streptomycin solution.

### 2.2. Peptides

All peptides were synthesized by ChinaPeptides, Inc (Shangai, China) using Fmoc chemistry. Peptides were provided at >95% purity, and the purity and structure were confirmed with RP-HPLC and ESI-MS.

### 2.3. Bioinformatics

Physiochemical properties of the peptides were calculated using the Antimicrobial Peptide Database (APD2) [23]. The percent hydrophobicity is defined as the ratio of hydrophobic residues to total residues.

### 2.4. Bactericidal Assays

The antimicrobial activity of the peptides against *M. smegmatis* was determined as previously described [20,21]. The antimicrobial activity for each peptide was determined through 3 h incubation time. The appropriate dilutions of each well were plated in triplicate and the extent of killing for the peptides were determined.

Briefly, 1 × 10^5^ CFU per well of bacteria were incubated with different peptide concentrations in a 50 μL solution of Buffer Q consisting of 10 mM potassium phosphate buffer at pH 7.2 and 0.1% MB7H9 (3 h, 37 °C). Serial dilutions were then prepared in Buffer Q and plated in triplicate on MB7H9 plates, which were incubated (37 °C, 24 to 48 h) and CFUs counted. Bacterial survival at each peptide concentration was calculated based on the ratio of the number of colonies on each experimental plate and the average number of colonies observed for assay cultures lacking peptides. The peptide concentration required to kill 50% of the viable *M. smegmatis* in the assay cultures (EC_50_) was determined by plotting percent killing as a function of the log of peptide concentration (log μg/mL) and fitting the data using GraphPad Prism 5 (GraphPad Software Inc., San Diego, CA, USA). EC_50_ values were determined by plotting percent killing as a function of the log of peptide concentration (log μg/mL), and fitting the data from the antimicrobial assays to a standard sigmoidal dose-response curve, using Equation (1), where Y corresponds to bacterial killing (%) at a given peptide concentration (μg/mL), with X being the logarithm of that concentration (log μg/mL). In the equation, “Top” and “Bottom” refer to the upper and lower boundaries, and were constrained to values <100% and >0%, respectively. For the purpose of graphing, samples that had no peptide are plotted at 10^−9^ μg/mL

Y = Bottom + (Top − Bottom)/(1 + 10^[(logEC50−X) × Hill Slope]^) (1)

Errors were reported based on the 95% confidence interval calculated on the log EC_50_ values to represent *p* < 0.05.

### 2.5. Minimum Inhibitory Concentration (MIC) Measurements

The MICs of the peptides and drugs against mycobacteria were determined by the broth microdilution method as described previously. All anti-mycobacterial activity evaluations were performed using MIC assays in MB7H9 broth with 10% oleic acid albumin dextrose complex (OADC) as previously described [24]. Briefly, a range of concentrations (0.97, 1.95, 3.9, 7.8, 15.6, 31.3, 62.5, 125, 250, 500 μg/mL) of the peptides was prepared by serial dilution and added to an equal volume of exponentially grown bacterial culture (100 μL) in each well of a 96-well plate. Microtiter plates were incubated for 48 h at 37 °C and scored as either growth or no growth. The MIC was defined as the concentration at which no microbial growth was observed visually or spectrophotometrically via readings of optical density (OD) at 600 nm (TECAN Safire2, Mannedorf, Switzerland). Growth media containing only microbial cells was used as the negative control. Each MIC test was carried out in six replicates for antibiotics and three replicates for antimicrobial peptides and repeated three times.

### 2.6. Checkerboard Assay

Antimicrobial interactions were determined by the checkerboard assay [25]. The antibiotic was diluted serially along the ordinate, while the antimicrobial peptide was diluted along the abscissa. First, two-fold serial dilutions of antibiotic rifampicin or polymyxin B and each antimicrobial peptide were prepared (ATRA-1A, hBD3-Pep4, mCRAMP, and LL37). Next, 50 μL of rifampicin and antimicrobial peptides were added into 100 μL of bacterial solution (containing approximately 10^5^ CFU mL^−1^) in each well of a 96-well plate. The plates were then incubated at 37 °C and read after 72 h for *M. smegmatis*. Assessment of microbial growth was done visually or spectrophotometrically via OD readings at 600 nm (TECAN, Mannedorf, Switzerland). The resulting checkerboard contains combination of antibiotics and antimicrobial peptides with wells that contain the highest concentration of each antibiotic at opposite corners. The fractional inhibitory concentration index (FICI) was calculated for each combination using this equation: FICI = FIC_A_ + FIC_B_, where FIC_A_ = MIC of drug A in combination/MIC of drug A alone, and FICB ¼ MIC of drug B in combination/MIC of drug B alone. FICI of ≤0.5 was interpreted as synergy, 0.5 < FICI ≤ 1.0 as additive, 1.0 < FICI ≤4.0 as indifferent, and FICI > 4.0 as antagonism [25].

### 2.7. Bacterial Cytoplasmic Membrane Depolarization Assay

**Figure 1:**
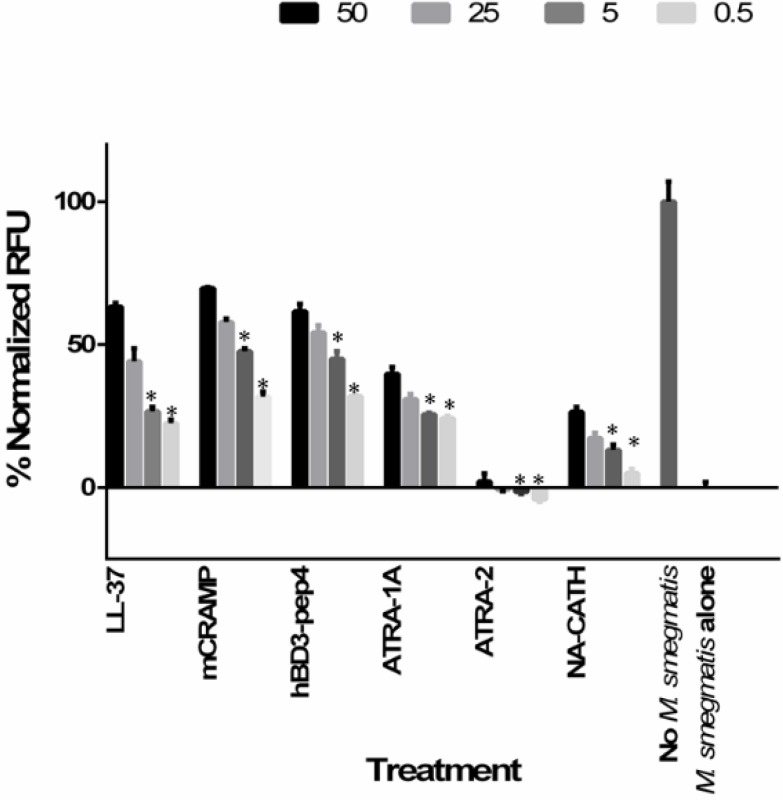
Membrane depolarization activity by LL-37, mCRAMP, hBD3-Pep4, ATRA-1A, ATRA-2, and NA-CATH. Depolarization determined using DiSC_3_(5) (50 μg/mL) at multiple concentrations of peptide (50, 25, 5, and 0.5 μg/mL). Experiments were performed three times with *n* = 2 each time; for each concentration of peptide all the data were combined (*n* = 6) for the graph and the analysis. Means ± the SD are shown; *: *p* ≤ 0.001.

Membrane depolarization assay was studied using DiSC_3_(5) as previously reported [13]. Depolarization of a membrane can be visualized by a drop in fluorescence. After interaction with intact cytoplasmic membrane, the fluorescent probe DiSC_3_(5) was quenched. After incubation with the antimicrobial peptide, the membrane potential was lost, and the probe was released to the medium, ensuing in an increase of fluorescence that can be quantified and monitored as a function of time. In brief, enumerated frozen bacteria were pelleted and washed twice in phosphate buffer (pH 7.2) and then resuspended to 4 × 10^7^ CFU/mL in phosphate buffer containing 50 μg/mL DiSC_3_(5). Then, 100 uLs of this suspension was added to wells of a black 96-well plate. The plate was incubated in a TECAN Safire2 spectrofluorometer and monitored until fluorescence leveled off. Next, 100 μLs of various concentrations of peptide in phosphate buffer (pH 7.2) were added to each well. Bacteria without peptide and peptide without bacteria served as controls. The plate was immediately returned to the spectrofluorometer. Readings were taken every 15 s for 5 min (excitation = 622 nm; emission = 670 nm). Peak relative fluorescent units (RFU) at each concentration are shown in Figure 1, and values were analyzed against wells with no peptide treatment and against peptide controls with a Student’s *t*-test.

### 2.8. Bacterial Cytoplasmic Membrane Permeation

**Figure 2:**
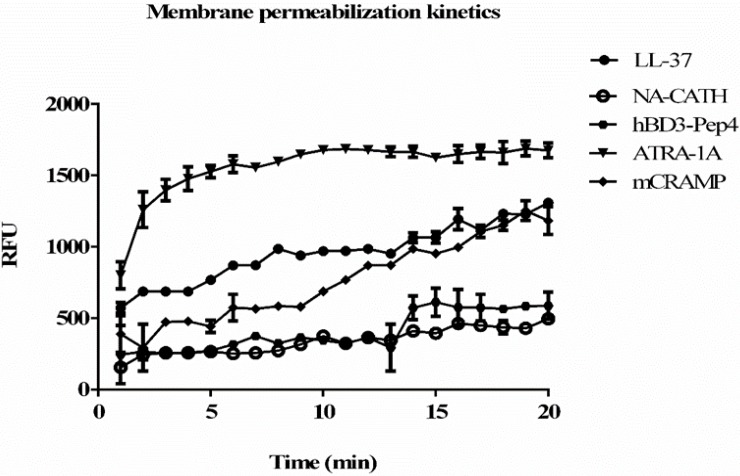
Kinetics of permeabilization and binding of ethidium bromide to DNA with 50 μg/mL of peptide concentration. Pore-forming activity by the peptides tested in the studies: hBD3-Pep4, ATRA-1A, mCRAMP, LL-37, and NA-CATH. An increase in fluorescence demonstrates greater binding of DNA by ethidium bromide, which indicates the formation of pores in the bacterial membrane. Permeablization of membrane after 20 min was compared. Experiments were performed in triplicate (*n* = 3). Means ± the SD are shown; *p* ≤ 0.01 when comparing each peptide activity at 20 min to untreated control.

Bacterial cytoplasmic membrane permeation is monitored by using the ethidium bromide uptake assay. The ethidium bromide uptake assay is performed as previously detailed with some modifications [13,26]. *M. smegmatis* is grown until log phase in MB7H9 (Difco) broth with 2% (*w*/*v*) glucose as the carbon source, and 0.05% (v/v) Tween 80 in a shaking incubator (37 °C). Bacteria is centrifuged, washed with PBS, and then adjusted to an OD 600 nm of 0.1 Phosphate Buffer. 180 μL of bacteria is added to 10 μL ethidium bromide (10 μM final concentration) and 10 μL peptide in various concentrations, the experiment was performed in triplicate. The plate is read in a Tecan Safire^2^ spectrofluorometer every 2 min for 30 min at 37 °C: (excitation = 540 nm, emission = 590 nm). Peak RFU at 20 min is used in Figure 2, and values were analyzed against wells with no peptide treatment with a Student’s *t*-test.

### 2.9. In Vitro Intracellular Activity in Infected Macrophages

The intracellular killing assay was performed as described previously [27], with the following modifications: 5 × 10^5^ J774A.1 cells are seeded in a 24-well plate, cells are allowed to adhere for 2 h. Cells are then infected with *M. smegmatis* mc^2^155 strains at multiplicity of infection of 10 in triplicate. After 4 h the supernatants were removed and adherent cells are washed with media. The cells are then treated with 20 μg/mL of gentamicin for killing of extracellular bacteria for one hour. Macrophages were treated with ATRA-1A, mCRAMP, hBD3-Pep4, LL-37 or with antibiotics rifampicin or polymyxin B. Macrophages were also treated with peptide drug combination to study the synergetic effect on intracellular killing. After appropriate incubation time, the cells were washed and lysis of cells is done by adding 0.5% Triton X100. The intracellular survival of *M. smegmatis* was determined by plating serially diluted culture on MB7H9 agar plates and the colonies were enumerated after three days.

## 3. Results

In this study, the antimicrobial activity of small, synthetic antimicrobial peptides was compared to standard antibiotics such as rifampicin, fosmidomycin, and polymyxin B, as well as with full-length peptides NA-CATH, mCRAMP, and LL-37 in terms of EC_50_ and antimycobacterial activity by minimum inhibitory concentration (MIC) against *M. smegmatis*.

### 3.1. Synthetic ATRA Peptides Exert Antimycobacterial Effects

**Figure 3:**
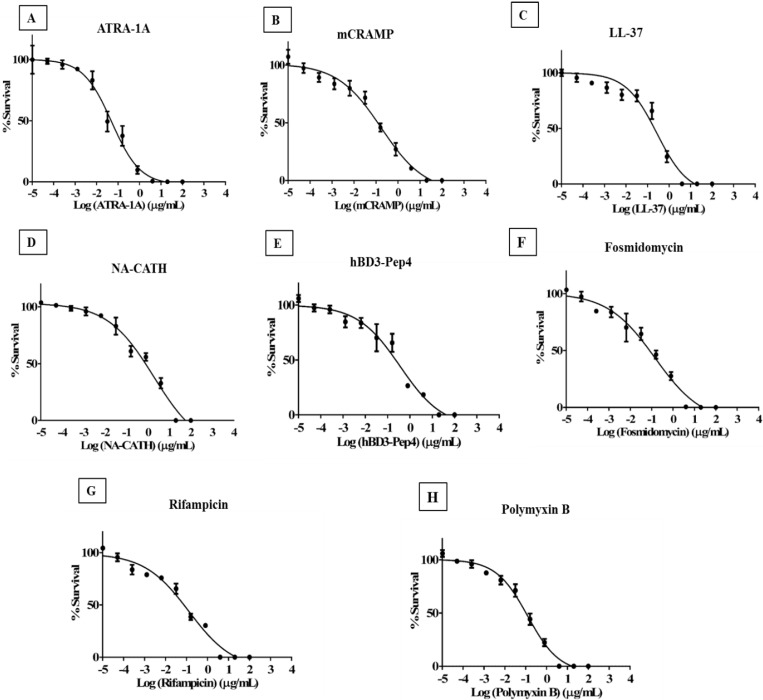
Effectiveness of antimicrobial peptides against *M. smegmatis*. Percent survival was calculated by CFU after 3 h incubation with various peptide concentrations in 10 mM of phosphate buffer (pH 7.4) supplemented with 0.1% of MB7H9 supplemented with OADC. (**A**) EC_50_ of ATRA-1A was found to be 0.05 μg/mL; (**B**) EC_50_ of mCRAMP was found to be 0.17 μg/mL; (**C**) EC_50_ of LL-37 was found to be 0.27 μg/mL; (**D**) EC_50_ of NA-CATH was found to be 1.88 μg/mL; (**E**) EC_50_ of hBD-3 Pep4 was found to be 0.36 μg/mL; (**F**) EC_50_ of Fosmidomycin was found to be 0.14 μg/mL; (**G**) EC_50_ of Rifampicin was found to be 0.13 μg/mL; (**H**) EC_50_ of polymyxin B was found to be 0.12 μg/mL.

*M. smegmatis* was subjected to a snake-derived helical cationic peptide, NA-CATH, and two short synthetic peptides derived from NA-CATH, ATRA-1A and ATRA-2 (Table 1, Figure 3) for their *in vitro* activity. These two ATRA peptides differ by two residues at the third (F/A) and 10th (L/P) position. As previously reported, ATRA-1A was highly effective as an antimicrobial peptide against *Francisella novicida* [20], *Pseudomonas aeruginosa* [21], and *S. aureus* [20], while ATRA-2 was ineffective. These two peptides have the same net charge of +8, a highly similar sequence, and the same length (11 residues). However, the potency of ATRA-1 is higher than ATRA-2, indicating that the sequence differences may influence activity of the peptides. The EC_50_ value of ATRA-1A against *M. smegmatis* was determined to be 0.05 μg/mL (Figure 3A), while the EC_50_ value for ATRA-2 did not cause significant killing of *M. smegmatis* at any concentration when tested in EC_50_ conditions. The antimycobacterial EC_50_ of ATRA-1A is equivalent to 0.035 μM (Table 2), making it the most effective peptide identified to date against this organism.

**Table 1:** Sequences and net charges of tested antimicrobial peptides. Short peptides contain a C-terminal carboxamide indicated by the italicized “-NH2”.

Peptide	Sequence	Net Charge at pH 7	Hydro-Phobicity
NA-CATH	**KRFKKFFKKLK**NSVK**KRAKKFFKKPK**VIGVTFPF (ATRA-1) (ATRA-2)	+15	38%
ATRA-1A	KR**A**KKFFKK**L**K-*NH2*	+8	36%
ATRA-2	KR**A**KKFFKK**P**K-*NH2*	+8	27%
hBD3-Pep4	RGRRSSRRKK*-NH2*	+7	0%
mCRAMP	GLLRKGGEKIGEKLKKIGQKIKNFFQKLVPQPEQ	+6	29%
LL-37	LLGDFFRKSKEKIGKEFKRIVQRIKDFLRNLVPRTES	+6	35%

**Table 2:** EC_50_ of peptides and antibiotics against *M. smegmatis*.

Peptide/Antibiotic	Molecular Weight (g/mol)	EC_50_			Fold *vs.* Rifampicin
		μg/mL	95% Confidence Interval	μM	
Rifampicin	822.94	0.13	0.061 to 0.276	0.159	1.0
Polymyxin B	1301.56	0.12	0.086 to 0.180	0.096	1.65
Fosmidomycin	183.1	0.14	0.062 to 0.326	0.780	0.20
NA-CATH	4175.26	1.88	0.622 to 5.68	0.451	0.354
hBD3-Pep4	1286.51	0.36	0.159 to 0.824	0.287	0.554
LL-37	7793.33	0.27	0.150 to 0.491	0.060	2.65
mCRAMP	3878.67	0.17	0.093 to 0.325	0.044	3.61
ATRA-1A	1420.84	0.05	0.034 to 0.075	0.035	4.54
ATRA-2	1404.80	>100	-	-	-

### 3.2. Cathelicidin Peptides mCRAMP, NA-CATH, and LL-37 Are Antimycobacterial

The antimycobacterial effectiveness of mCRAMP, the mouse cathelicidin, NA-CATH (Chinese cobra derived cathelicidin), and LL-37 human cathelicidin was tested against *M. smegmatis*. The EC_50_ of mCRAMP was determined to be 0.17 μg/mL (0.044 μM) (Figure 3B), which is much lower than the reported value of Carlos Santos *et al.*, who reported an EC_50_ value of 58.81 μM against *M. avium* [28], suggesting that this a physiologically relevant potency of mCRAMP. The EC_50_ of the LL-37 was determined to be 0.27 μg/mL (0.06 μM) (Figure 3C), which agrees with the published sensitivity *of M. smegmatis* to LL-37 [9] and of *M. tuberculosis* to LL-37 [29]. Previously published results for *M. tuberculosis* showed a 65% killing of *M. tuberculosis* with 100 μg/mL of LL-37 [30]. The EC_50_ of NA-CATH (Figure 3D) was determined to be 1.88 μg/mL, which is higher than the EC_50_ value obtained for ATRA-1A. The hemolytic activity of the peptides ATRA-1A, NA-CATH, and LL-37 was determined previously by our group [19]. NA-CATH and ATRA-1A did not elicit statistically significant hemolysis compared to PBS (pH 7) [22]. mCRAMP is also reported to have no hemolytic activity [31].

### 3.3. Human *β*-Defensin Derivative hBD3-Pep4 Exerts Antimycobacterial Activity

We also tested the activity of Peptide 4 of hBD3 [2] and found that it has potent antimicrobial activity against *M. smegmatis* with an EC_50_ of 0.36 μg/mL (Figure 3E). This very short peptide has an EC_50_ value of 0.287 μM (Table 2). This result was exciting, as the activity exceeded the reported value of other human beta-defensins tested. Corrales-Garia *et al.* reported activity of hBD2 of 1.5 μM against H37RV and 6.8 μM of hBD3- M-hBD2 [32,33]. Human β-defensin is reported to be a low hemolytic and did not elicit significant hemolysis activity in comparison to PBS [34].

In summary, the EC_50_s of various antimicrobial peptides were determined against *M. smegmatis*. The most effective peptide by EC_50_ was ATRA-1A at 0.035 μM. mCRAMP was the second most effective at 0.04 μM. The peptide hBD3-Pep4 was effective at 0.287 μM. ATRA-2, the variant peptide with a substitution of the amino acid F→P, was absolutely ineffective against *M. smegmatis*. We also tested the EC_50_s of various antibiotics as controls with tested peptides; the observed EC_50_s for the antibiotics were 0.096, 0.78, and 0.159 μM for polymyxin B, fosmidomycin and rifampicin, respectively (Figure 3F–H).

### 3.4. Minimum Inhibitory Concentration of Peptides to Inhibit Mycobacterial Growth

While the EC_50_ experiments are typically carried out in 10 mM phosphate buffer with pH 7.2 for 3 h [6,20], the Minimal Inhibitory Concentration (MIC) is determined in bacterial broth (resulting in a higher salt concentration) with a 48 h incubation. We confirmed the sensitivity of *M. smegmatis* to common antibiotics such as polymyxin B, rifampicin, and fosmidomycin [35,36,37]. The MIC for the human cathelicidin LL-37 was found to be 31.8 μg/mL (Table 2), which was considerably higher than the reported value of 5 μg/mL against *M. tuberculosis* [30]. The reported MIC of full-length hBD3 peptide against *M. tuberculosis* is between 24 and 96.2 μg/mL [33], which agreed with the obtained MIC against *M. smegmatis* of 62.6 μg/mL. Recently, bovine neutrophil beta-defensin 5 was also found to be antimicrobial against *M. smegmatis* in MIC assays [38]. In a different assay format, protegrin and rabbit and human defensins were also found to be active against *M. tuberculosis* [39]. By MIC assay, the small synthetic peptides tested (ATRA-1A, hBD3-Pep4) inhibited growth of *M. smegmatis*, with most of them showing activity at low microgram concentrations of 31.3 and 62.6 μg/mL respectively, as depicted in Table 3. ATRA-1A had the same MIC as LL-37 by μg/mL, but was less effective than LL-37 on a molar comparison basis, while hBD3-Pep4 was about half as effective as ATRA-1A on a μg/mL and a molar comparison basis. ATRA-2 was the most ineffective peptide with an MIC of >125 μg/mL. A similar approach was published by Ramon-Garcia *et al.*, who showed MIC activity with a set of completely synthetic random small cationic peptides [29]. We used antibiotic fosmidomycin as one of the control antibiotics for our study, which was found to be highly effective against *M. smegmatis*, consistent with its anti-*M. tuberculosis* activity [35,36,37]. Thus, even in the high-salt conditions of the MIC assay (often associated with lack of activity of antimicrobial peptides such as LL-37), the two short, synthetic peptides ATRA-1A and hBD3-Pep4 both showed significant anti-*M. smegmatis* activity.

**Table 3:** Minimum inhibitory concentrations (MIC) of peptides against *M. smegmatis*.

Peptide/Antibiotic	MIC (μg/mL)	MIC μM	Fold *vs.* Rifampicin (μM)
Rifampicin	3.9	4.73	1
Fosmidomycin	7.8	1.86	0.39
Polymyxin B	7.8	5.99	1.26
mCRAMP	15.6	4.02	0.85
LL-37	31.3	4.01	0.85
ATRA-1A	31.3	22.01	5.11
hBD3-Pep4	62.6	48.65	10.28
NA-CATH	250	59.80	12.64
ATRA-2	>125	-	-

### 3.5. Membrane Permeablization and Depolarization by Peptides

The promising preliminary results encouraged us to further investigate the mechanisms of action of peptides at the bacterial cell level. The membrane depolarization activity was measured with the fluorescent chemical DiSC_3_(5), which is sensitive to the polarization of membranes. Depolarization of bacterial membranes indicates very small, transient pore formation, which allows damaged membranes to leak ions and interferes with proton motive force and other gradients that store chemical energy. As depicted in Figure 1, within 1 min it was observed that peptides mCRAMP, hBD3-Pep4, and LL-37 were able to depolarize at values >70%, significantly increasing their permeabilization of mycobacterial cells compared to the peptide ATRA-1A. NA-CATH was able to depolarize at a value of >35%. mCRAMP, hBD3-Pep4, and LL-37 depolarize the *M. smegmatis* membrane at concentrations as low as 0.5 μg/mL (*p* < 0.001), representing a dose-dependent response to peptide concentration (*i.e.*, depolarization by LL-37 at 0.5 μg/mL is statistically different than depolarization by 50 μg/mL as analyzed by ANOVA). ATRA-1A and NA-CATH also permeablized the membrane but showed slower kinetics (Figure 1). ATRA-2 did not depolarize membranes, which can be the reason for it being ineffective against *M. smegmatis*.

These results suggest that ATRA-1A, mCRAMP, hBD3-Pep4, and LL-37 peptides are capable of quickly forming pores in the *M. smegmatis* membrane and suggest this is the mechanism by which these peptides kill bacteria.

When ethidium bromide uptake assay was performed (Figure 2), it was found that peptide ATRA-1A presented the best permeabilization at the concentration of 50 μg/mL and has faster kinetics than the other peptides. However, mCRAMP and LL-37 peptides showed a faster increase in permeablization than hBD3-Pep4 and NA-CATH, but were slower than ATRA-1A (Figure 2). With ATRA-1A the fluorescence gradually increased over the 20 min experimental time frame. When the extent of the permeabilization was examined at 20 min, ATRA-1A had exerted the most permeabilization, with LL-37 and mCRAMP also being very efficient. Overall, this data suggests that ATRA-1A permeabilizes *M. smegmatis* rapidly and effectively.

### 3.6. Synergy with Antibiotics

Various combination therapies of antibiotics and AMPs examined in this study were evaluated for improving the effectiveness of the antibiotics and preventing or delaying the development of antibiotic resistance. Combination therapy for antimicrobial peptides ATRA-1A, hBD3-Pep4, and mCRAMP has not been reported in literature for *Mycobacterium* spp. Synergy with antibiotics may reduce costs and reduce the peptide toxicity of the treatment for *Mycobacterium* infections, similar to other peptide-antibiotic combinations reported previously [16,27].

Cationic peptides are considered to be promising antibiotic candidates due to the fact that these peptides are less likely to develop drug resistance and have broad spectrum activity. However, the clinical usage is limited due to poor potency, specificity, and *in vivo* stability [40]. To overcome these shortcomings as a synergistic effect between peptides and traditional antibiotics could reduce the dose of each drug in combination, prevent drug resistance, and result in a greater antibacterial effect than the sum of the effects due to single agents [41]. Rifampicin has been reported to act synergistically when administered in conjunction with anti-microbial peptides, possibly as a consequence of the peptide-mediated membrane disruption, which in turn enhances uptake of the drug [42]. Khara *et al.* have shown that in combination with rifampicin, the peptide M(LLKK)2M has been shown to delay the emergence of rifampicin resistance [27]. Kalita *et al.* have previously reported the synergistic effect of HNP-1 and rifampicin in a macrophage model. They reported a remarkably significant reduction in bacterial load in the organs of infected mice by the synergy [43]. To determine the interactions between the short cationic peptides and rifampicin, synergy studies were performed using the checkerboard assays. We tested if there was any observable synergy between these active small peptides and the commonly used antibiotics rifampicin and polymyxin B. The antibacterial interactions between the antimicrobial peptides LL-37, ATRA-1A, mCRAMP, hBD3-Pep4 and antibiotics rifampicin and polymyxin B were analyzed.

As shown in Table 4, LL-37 and mCRAMP displayed synergism with rifampicin with an FICI value of 0.32 and 0.35, respectively. The peptide-drug combination of ATRA-1A and rifampicin has an additive effective with an FICI value of 0.56. Notably, a low peptide concentration equivalent to one-eighth of its MIC halved the amount of rifampicin required to inhibit *M smegmatis* growth in the cases of ATRA-1A and mCRAMP. Similar levels of synergy were also observed with polymyxin B and antimicrobial peptides LL37, ATRA-1A, hBD3-Pep4, and mCRAMP with FICI values of 0.37, 0.37, 0.37, and 0.5 respectively. Thus, polymyxin B was able to show synergistic activity with both the small, synthetic peptide ATRA-1A, hBD3-Pep4, and full-length cathelicidins. Although there are various reports of combination studies suggesting the synergistic effect of antimicrobial peptides and rifampicin, the present data is the first to report the synergistic effects of ATRA-1A and hBD3-Pep4 with polymyxin B.

**Table 4:** Checkerboard assay of antibiotics and antimicrobial peptides against *M. smegmatis*. Rifampicin/polymyxin B and ATRA-1A/LL-37/mCRAMP. Synergy is defined as FICI ≤ 0.5, 0.5 < FICI ≤ 1.0 as additive, 1.0 < FICI ≤ 4.0 as indifferent, and FICI > 4.0 as antagonism [25]. Synergism was observed for several of the peptide drug combinations (shaded). Experiments were performed in triplicate (*n* = 6). Means ± the SD are shown; *p* ≤ 0.001.

Drug Combination	MIC (μg/mL)		FIC	FICI
	Alone	Combined		
Rifampicin ATRA-1A	3.9	1.95	0.5	0.56
	31.3	3.9	0.06	
Rifampicin hBD3-Pep4	3.9	1.95	0.5	0.56
	62.6	3.9	0.06	
Rifampicin LL-37	3.9	0.97	0.25	0.32
	31.3	3.9	0.12	
Rifampicin mCRAMP	3.9	0.97	0.25	0.35
	15.6	1.95	0.12	
Polymyxin B ATRA-1A	7.8	1.95	0.5	0.37
	31.3	3.9	0.12	
Polymyxin B hBD3-Pep 4	7.8	1.95	0.25	0.37
	62.6	7.8	0.12	
Polymyxin B LL-37	7.8	1.95	0.25	0.37
	31.3	3.9	0.12	
Polymyxin B mCRAMP	7.8	1.95	0.25	0.5
	15.6	3.9	0.25	

### 3.7. Intracellular Killing of Mycobacteria Peptide-Treated Macrophages

**Figure 4:**
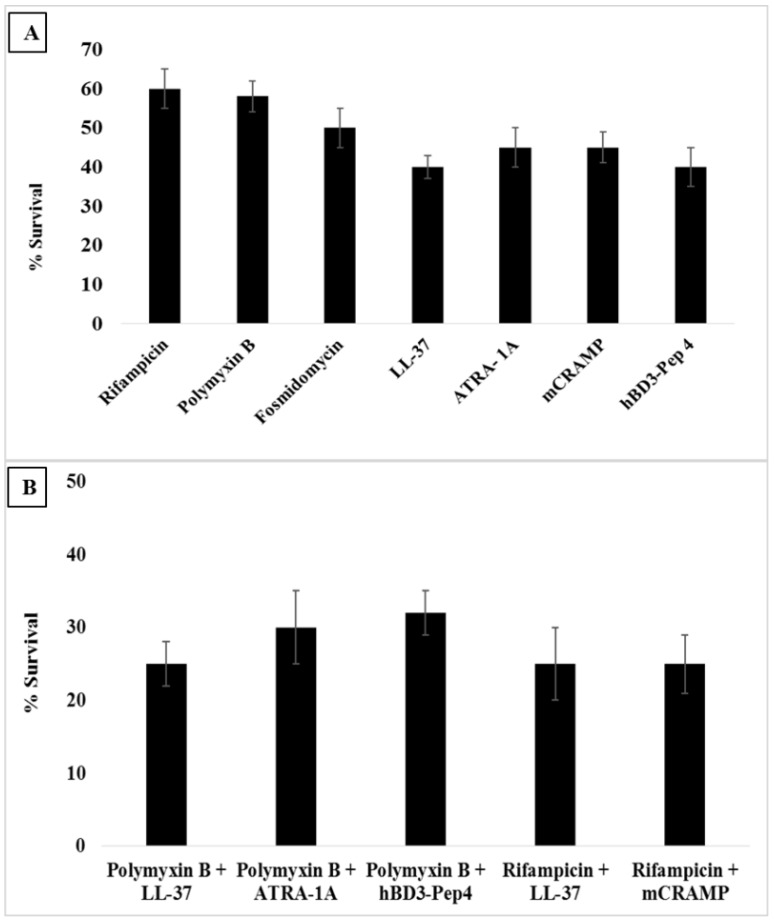
Intracellular killing of mycobacteria in macrophages. Macrophages were infected with *M. smegmatis* for 12 h and then incubated with antibiotics, antimicrobial peptides, and peptide-drug combinations. Macrophages infected with bacteria alone were used as a control. Cells were lysed, and bacterial intracellular survival was determined by CFU assay. (**A**) Intracellular survival of *M. smegmatis* after treatment with antibiotics or antimicrobial peptides; *p* ≤ 0.05 significantly different compared to untreated; (**B**) Intracellular survival of *M. smegmatis* after treatment with peptide-drug combination. Experiments were performed in triplicate (*n* = 3). Means ± the SD are shown; *p* ≤ 0.05 significantly different compared to untreated.

To test the intracellular survival of mycobacteria, *M. smegmatis*-infected macrophages were treated with 10 μg of either antibiotics rifampicin, polymyxin B, or fosmidomycin, or with peptides LL-37, ATRA-1A, hBD3-Pep4, or mCRAMP. As shown in Figure 4A, from the tested short synthetic peptides, hBD3-Pep4 was found to be potentially effective, killing 60% of mycobacteria compared to mCRAMP or ATRA-1A, which showed a killing efficiency of 55%. We found 60% killing of *M. smegmatis* when tested with LL-37. Sonawane *et al.* have also reported the 50% of killing with 10 μg/mL of LL-37 [9]. hBD3-Pep4 was found to be equally efficient as LL-37.

Combination therapy of antimicrobial peptides and antibiotics was found to be very efficient in eliminating intracellular *M. smegmatis*. To test the intracellular killing with the peptide and drug combination, *M. smegmatis*-infected macrophages were treated with 10 μg of a peptide-drug combination. The intracellular killing increased in combination therapy than that observed in individual AMPs or antibiotics. As depicted in Figure 4B, the synergetic effect of polymyxin B and LL-37, and rifampicin and LL-37 showed 75% killing. However, 75%, 70%, and 68% killing was observed with rifampicin and mCRAMP, polymyxin B and ATRA-1A, and polymyxin B and hBD3-Pep4, respectively. We did not check the intracellular survival of *M. smegmatis* for a prolonged period of time after infection because previous findings have shown that macrophages efficiently kill *M. smegmatis* after 12 h of infection and that, after 24 h, the majority of the bacteria are cleared by macrophages [9]. It has been previously reported for non-pathogenic *M. smegmatis* that the macrophage needs at least 4 h and up to 24–48 h after infection to completely kill the bacteria [44].

## 4. Discussion

Antimicrobial peptides such as cathelicidin and defensins have a clearly important role in pulmonary tuberculosis. The possible induction of cathelicidin expression through vitamin D treatment [45,46,47,48] further supports this concept. Treatments for tuberculosis may involve the development of shorter, synthetic peptides with improved activity or stability and lower cost. The present study demonstrates that several cationic AMPs were effective against *M. smegmatis*. *M. smegmatis* closely resembles *M. tuberculosis*, but it is non-pathogenic and has a shorter doubling time than *M. tuberculosis*, making it safe and practical to culture in the laboratory. Both the species show similarities at various levels including similar reactions to acid-fast staining, similar cell wall structures, and both of them synthesize mycothiol and are capable of forming biofilms. High levels of similarities have also been reported in the genes between the two species [49,50,51,52]. These similarities in *M. smegmatis* make it a safe and practical model to screen for probable drug candidates for tuberculosis [53,54].

Cathelicidins are identified based on a conserved N-terminal domain, the cathelin domain, present in the inactive precursor peptide [55]. The cathelicidin peptides tested in this study were, as a class, generally active against *M. smegmatis.* The active human cathelicidin antimicrobial peptide LL-37 is derived *in vivo* by proteolysis from the C-terminal end of the human CAP18 protein (hCAP18) [56]. LL-37 is a 37-residue cationic peptide that forms alpha-helical structures when in association with the bacterial cell membrane [7,56], a critical component of the antimicrobial mechanism employed by LL-37 and other helical cathelicidins. LL-37 has been shown to be antimicrobial against both Gram-positive [22] and Gram-negative [21] bacteria, and has even been found to play a host-directed role in wound healing [57]. LL-37 is shown to have a host-directed chemotactic effect on inflammatory cells, while at the same time playing a broader role in immunomodulation in systemic settings such as the lung, and has been reported to play a major role in protecting humans against naturally occurring respiratory diseases [58]. Thus, the demonstration here and in published studies [9] of *in vitro* activity of LL-37 against *M. smegmatis* suggests that this peptide may be of interest for further study. In further support of the activity of cathelicidin peptides against *M. smegmatis*, the mouse cathelicidin mCRAMP as well as the snake cathelicidin NA-CATH were shown to be active. These results suggest that the helical, amphipathic, cationic cathelicidin peptides may be effective as a class of peptides against this organism. Data recently reported in the literature suggests that similar results may be found against *M. tuberculosis* [9,30,59].

In contrast, ATRA-2 did not kill *M. smegmatis* at any concentration. This agrees with our previous findings that the amino acid substitutions made to produce ATRA-2 rendered it ineffective as an AMP against most bacteria [19,20,21,22]. The introduction of a proline at residue 11 likely disrupts the helical structure of this peptide, thus interfering with its antimicrobial activity [19].

With regard to potential treatments, it would be advantageous to have a peptide that is smaller than the cathelicidin peptides, as they are quite expensive to synthesize. In particular, two small, synthetic peptides were identified with high levels of antimicrobial activity against *M. smegmatis*. One synthetic peptide, ATRA-1A, is inspired by a cathelicidin peptide NA-CATH, from the Chinese king cobra, *Naja atra.* The NA-CATH peptide demonstrates broad-spectrum antibacterial activity against both Gram-positive and Gram-negative bacteria [19,20,21,22] and was found in this study to be active against *M. smegmatis*. Our previous studies have confirmed the amphipathic and highly helical nature of ATRA-1A [19], thus suggesting that this peptide shares the general properties of being cationic, amphipathic, and helical with the cathelicidins [30]. The second synthetic peptide, hBD3-Pep4, is a small peptide inspired by the human beta-defensin peptide hBD3 [2,60]. Human genomic sequences have revealed the existence of five beta-defensin gene clusters containing approximately 30 known and potential beta-defensin genes [61,62]. These peptides demonstrate antimicrobial effectiveness against a broad spectrum of Gram-positive and Gram-negative bacteria, fungi, and some enveloped viruses, with hBD-3 demonstrating the broadest antimicrobial effectiveness. While beta-defensins are capable of directly exerting antimicrobial activity against invading pathogens, they also demonstrate chemotactic properties, thus bridging innate immunity and adaptive immunity [16,30,63]. There have not been many reports for an *in vivo* role for hBD3 against *Mycobacterium* spp.; however, *in vitro* activity has been demonstrated [33]. This peptide is not as active as the ATRA-1A peptide. However, this peptide could be of interest for further testing or development. Interestingly, the beta-defensin peptides are not primarily helical, although they are cationic and amphipathic as a class.

Finally, we tested if there was any synergy between these active small peptides and the commonly used antibiotic rifampicin. Recently, such a synergy was reported for synthetic peptides with the sequence M(LLKK)_2_M [27]. Peptide-mediated destruction of membrane integrity may facilitate the entry of antibiotics to cytoplasmic targets and is suggested to be responsible for the observed synergy. We sought to determine whether such synergy could be more broadly applicable to our two short, synthetic peptides against *M. smegmatis*. However, significant synergy with rifampicin was only observed for the full-length cathelicidins LL-37 and mCRAMP and not for the short, synthetic peptides ATRA-1A and hBD3-Pep4. Both the short peptides showed synergistic activity with the cyclic peptide antibiotic polymyxin B, as did LL-37. mCRAMP synergized with Polymyxin B with a FICI score of 0.5. These results showing the activity of two small, synthetic peptides against *M. smegmatis* may provide a foundation for further research and development of these and other synthetic peptides for potential future use against *M. tuberculosis* [29].

Among the antimicrobial peptides tested for intracellular killing, hBD3-Pep4 had remarkable intracellular killing ability against *M. smegmatis* compared to conventional antibiotics like rifampicin and polymyxin B. However, when tested in combination therapy, hBD3-Pep4 and polymyxin B showed 68% killing in comparison to the 75% of polymyxin B and LL-37, rifampicin and LL-37, and rifampicin and CRAMP.

## 5. Conclusions

In this study, a series of short, synthetic, cationic peptides have been evaluated for their antimycobacterial activities. The peptides ATRA-1A, hBD3-Pep4, mCRAMP, NA-CATH, and LL-37 showed efficient activity against *M. smegmatis*. The peptides eradicate bacteria based on a membrane-lytic mechanism, and displays a synergistic interaction with rifampicin against *M. smegmatis*. The direct killing of bacilli by antimicrobial peptides as well as the increased penetration of peptides into the mycobacterial cells present in the macrophages make antimicrobial peptides potential candidates for further studies with *M. tuberculosis*.
